# The Association between Potential Exposure to Magazine Ads with Voluntary Health Warnings and the Perceived Harmfulness of Electronic Nicotine Delivery Systems (ENDS)

**DOI:** 10.3390/ijerph15040575

**Published:** 2018-03-23

**Authors:** Ce Shang, Scott R. Weaver, Nahleen Zahra, Jidong Huang, Kai-Wen Cheng, Frank J. Chaloupka

**Affiliations:** 1 Health Policy Center, Institute for Health Research and Policy, University of Illinois at Chicago, Chicago, IL 60608, USA; kwcheng@uic.edu (K.-W.C.); fjc@uic.edu (F.J.C.); 2 School of Public Health, Georgia State University, Atlanta, GA 300303, USA; srweaver@gsu.edu (S.R.W.); jhuang17@gsu.edu (J.H.); 3 Department of Economics, University of Illinois at Chicago, Chicago, IL 60608, USA; nahleen@gmail.com; 4 Division of Health Policy and Administration, School of Public Health, University of Illinois at Chicago, Chicago, IL 60608, USA

**Keywords:** ENDS, e-cigarettes, voluntary warnings, risk perception, magazine ads

## Abstract

(1) Background: Several brands of electronic nicotine delivery systems (ENDS) carry voluntary health warning messages. This study examined how potential exposure to ENDS magazine ads with these voluntary health warnings were associated with the perceived harmfulness of ENDS. (2) Methods: Risk perception measures and self-reported exposure to ENDS ads were obtained from the 2014 Georgia State University (GSU) Tobacco Products and Risk Perceptions Survey of a nationally representative sample of U.S. adults. We examined the association between potential exposure to magazine ads with warnings and the perceived harms of ENDS relative to cigarettes, using binary logistic regressions and controlling for general ENDS ad exposure and socio-demographic characteristics. (3) Results: Potential exposure to ENDS magazine ads with warnings was associated with a lower probability of considering ENDS to be more or equally harmful compared to cigarettes, particularly among non-smokers (OR = 0.16; 95% CI: 0.04–0.77). In addition, ad exposure, ENDS use history, race/ethnicity, gender, education, and income were also associated with harm perceptions. (4) Conclusions: This study did not find evidence that magazine ads with warnings increased misperceptions that ENDS are equally or more harmful than cigarettes. With more ENDS advertisements carrying warnings, more research is needed to determine how the warnings in advertisements convey relative harm information to consumers and the public.

## 1. Introduction

Health warnings are considered to be an effective method to inform consumers about the risks of smoking, and thereby reduce cigarette consumption [1,2]. However, with the increasing popularity of electronic nicotine delivery systems (ENDS) or e-cigarettes in the United States, the relative and absolute harms of ENDS use has become a subject of heated debate in the public health community [3]. From a continuum of risks perspective, ENDS are likely substantially less harmful than cigarettes [4], potentially offering health benefit to those who completely substitute ENDS for smoking cigarettes [5]. However, the long-term effects of ENDS and their overall public health impact remain unclear [6]. As a result, how health warning messages for ENDS are framed and conveyed to consumers is critically important, and may impact the harm perceptions and use of ENDS and cigarettes.

In May 2016, the U.S. Food and Drug Administration (FDA) issued a deeming rule that expands its regulatory authority to ENDS [6]. By August 2018, the deeming rule will require all ENDS advertisements and packages to carry the warning statement, “WARNING: This product contains nicotine. Nicotine is an addictive chemical”. This statement is required to occupy at least 20% of an advertisement (30% of the principal display panels of the package) and appear in at least 12 point font size. Some ENDS and e-liquid products had already carried voluntary warnings in their advertisements or on product packaging prior to issuance of the final deeming rule [7,8,9,10,11]. However, none of the voluntary warnings meet the font size and placement requirements in the deeming rule, and not all warnings mention nicotine or its addictiveness [7,8,10].

The requirement of an addiction warning for ENDS signifies the important role of harm and addiction beliefs in the use of tobacco products. However, evidence on the effectiveness of ENDS warnings in shaping harm or addiction perceptions and tobacco use is mixed. According to one focus group study, the FDA addiction warning has limited impact on tobacco users who already know nicotine is addictive, but it might inform young non-users [12]. However, some experts in tobacco warning research are concerned about whether this warning resonates with young people [13]. In general, expert opinions and focus group interviews show that modified risk or reduced risk statements that claim ENDS to be less harmful may increase ambiguity about risks, particularly among nonsmokers [13,14,15,16]

In addition to the aforementioned qualitative studies, studies using experimental methods have also led to mixed results. In general, when ENDS warning messages are presented to participants without being embedded in a print advertisement, they tend to raise harm or addiction perceptions and reduce preference for ENDS [17,18,19,20,21,22,23]. However, when ENDS warnings are embedded in an advertisement, the effects of warnings on harm or addiction perceptions and use intentions appear inconsistent, and vary by the warning placement, total size, font size, color, and content [21,24,25,26]. One study used heat maps to show that if warnings were placed in a similar way as the existing voluntary warnings (e.g., at the bottom of ads, small font sizes), participants would pay little attention to these warnings [21].

Nonetheless, beliefs and perceptions of ENDS harmfulness relative to cigarettes may be influenced by many factors. Recent research has shown that the proportion of U.S. adults who believed that ENDS are equally or more harmful compared to cigarettes has increased over time [27,28]. Nonsmokers, compared to smokers, are more likely to perceive ENDS to be equally or more harmful compared to cigarettes [27,29]. Regardless of smoking status, a significant proportion of US adults mistakenly believed that nicotine is the primary disease-causing chemical [30]. Studies also found that news articles discussed potential harms or risks of ENDS more often than their potential benefits [31], which may increase beliefs about ENDS harms while reducing beliefs about benefits [32]. Given the mixed evidence on ENDS warning messages and the complexity surrounding the perceptions of ENDS, it is critically important to assess how consumers may interpret messages from the FDA addiction warning and manufacturers’ voluntary warnings, and to what extent these messages may influence consumers’ harm perceptions about ENDS.

This study uses multivariate analyses to examine the association between potential exposure to ENDS magazine ads with voluntary warnings, and the relative harm perceptions of ENDS compared to cigarettes. In addition, the associations between ad exposure across all platforms, and demographic characteristics and harm perceptions, are also assessed. Since ENDS magazine ads reach consumers regardless of their tobacco use status and are dominated by the ads from the big ENDS manufacturers, which tend to include various voluntary warnings in the ads (including the proposed FDA addiction warning) [7], they pose a unique opportunity to explore how the FDA addiction warning and other warnings may be associated with harm perceptions. To our knowledge, this study is the first one to explore the potential exposure to ENDS ads with voluntary warnings in a non-experimental setting. Specifically, to identify the associations, this study capitalizes on the geographic variation in ENDS ads exposure reported in a large, national survey, and the significant variations in voluntary warnings in ENDS magazine ads in 2014 after the proposed deeming rule was issued [7].

## 2. Materials and Methods

### 2.1. Kantar Media and the Trinkets and Trash Surveillance System

Following the method outlined in Shang and Chaloupka (2017), we defined the voluntary warnings in ENDS magazine ads as those that described nicotine, its addictiveness, or its potential health risks (see Appendix A for warning statement examples) [7]. In March–November 2014, ENDS magazine ads captured by Kantar Media and the T&T surveillance system were evaluated. Kantar Media documented all paid advertising spaces and ad expenditure from the Publishers Information Bureau, Inc. (PIB) (New York, NY, USA) [23,24], with a coverage of all consumer magazines with more than 350 subscribers. Kantar Media also provided information on the size of the ad, units, and the percentage of the publication’s total circulation reached by the ad. Trinkets and Trash (T&T) surveillance system is a surveillance system that monitors and archives tobacco products and tobacco marketing activities, such as advertisements, direct mailings, e-mails, sweepstakes, coupon promotions, and brand websites, for research and educational purposes [33]. T&T archives ads of ENDS, including those published in magazines. A dichotomous variable was used to measure whether an ad carried voluntary warnings. The trend of voluntary warnings in ENDS magazine ads was then measured by aggregating the dichotomous variable to a three-month moving average, and weighted using ad unit, national equivalence, and expenditure [7]. This trend measures ENDS warnings in magazine ads at the national level by month in 2014. 

### 2.2. Tobacco Products and Risk Perceptions Survey (TPRPS)

In June–November 2014, the 2014 Tobacco Products and Risk Perceptions Survey was administered to a national probability sample of 5717 U.S. adults from an online panel, including a representative oversample of pre-identified cigarette smokers. The TPRPS is an annual, cross-sectional survey of a probability sample drawn from GfK’s KnowledgePanel, a probability-based web panel designed to be representative of non-institutionalized U.S. adults [27,34]. A final stage completion rate of 74.4% and a cumulative response rate of 6.6% were obtained. Post-stratification weights computed using an iterative proportional fitting (raking) procedure, using demographic and geographic distributions from the 2014 March Current Population Survey, as benchmarks, to adjust for sources of sampling and non-sampling error such as panel recruitment non-response and panel attrition, survey non-response, and oversampling of smokers, were applied. Warning trends from June to November 2014 were linked with the survey data using the month identifier. 

### 2.3. Constructs

#### 2.3.1. Harm Perception Outcome

In the Tobacco Products and Risk Perceptions Survey, the participants were provided with preamble text describing e-cigarettes, including the different terminology to refer to them, and pictures of different e-cigarette product types. Perceived harmfulness of ENDS relative to cigarettes was measured directly using the question: “Is using e-cigarettes less harmful, about the same level of harm, or more harmful than smoking regular cigarettes?” [35,36]. Participants could select from the above three statements and “I don’t know”. Using the responses, we constructed a dummy variable, which assigns those who reported to perceive ENDS to be equally or more harmful compared to cigarettes with 1, those who reported that ENDS are less harmful than cigarettes with 0. Those who reported that they don’t know (*N* = 1987) were dropped from the analyses.

#### 2.3.2. Potential Exposure to ENDS Magazine Ads with Warnings

Ever exposure to ENDS advertising in the TPRPS was assessed using the following question: “In which of the following places, if any, have you seen or heard advertisements for e-cigarettes?” Respondents were asked to select all places that applied, including magazines, newspapers, television, radio, internet, stores (inside or outside), and some other place (specify). The response could also be “I have not seen or heard advertisements for e-cigarettes anywhere”. A dichotomous variable “seeing any ads” was constructed by assigning those who reported that they have not seen/heard ads anywhere with 0, and those who reported any exposure with 1. 

Because these measures assess ever exposure to ENDS ads in magazines (or anywhere), we aggregated magazine ENDS ad exposure to the state-level average to reflect ENDS marketing by locations, which may be a better proxy for ENDS magazine ad exposure in 2014. Next, we multiplied this state-level average exposure to magazine ENDS ads by the proportion of magazine ads that carry voluntary warnings (i.e., the national-level warning trend measured using a three-month moving average described in Section 2.1) in 2014. 

The final and key measure is this product of the average exposure to magazine ENDS ads and the proportion of magazine ENDS ads with warnings, which reflects the potential exposure to ENDS magazine ads with warnings at the state level, and is a fraction between 0 and 1. 

#### 2.3.3. Other Covariates

We identified current smokers as those who had smoked at least 100 cigarettes, and were currently smoking every day or some days. E-cigarette ever use status was identified using a question that asked respondents to self-report whether they have ever tried e-cigarettes/electronic vapor products, even just one time. The surveys also provide detailed information on socio-demographic characteristics, based on which we constructed the following variables: gender, age, race/ethnicity (White, non-Hispanic as the omitted category; Black; non-Hispanic, other race; non-Hispanic, multi-race; non-Hispanic; and Hispanic), marital status (being married), household size, number of children under 18 in the household, educational attainment (less than high school as the omitted category, high school, some college, and Bachelor’s degree or higher), annual household income (≤$29,999 as the omitted category, $30,000–$74,999, and ≥$75,000), and employment status (unemployed as the omitted category, employed, and not in the labor force). 

### 2.4. Analysis

The associations between the potential exposure to magazine ENDS ads with voluntary warnings and harm perceptions were analyzed using weighted logistic regressions, after controlling for the correlates. Odds ratios and corresponding 95% confidence intervals were reported. Because prior research suggests that ENDS risk perceptions may depend on tobacco use history [27,28] we also conducted regressions stratified by whether respondents are currently smoking cigarettes. The final analytical sample consists of 3642 adult respondents, among whom 865 were cigarette smokers and 2777 were nonsmokers. We also conducted additional analyses to include respondents who answered “Don’t know” to the harm perception question, because they may differ from respondents who have formed perceptions in terms of ENDS use and susceptibility [27,37]. Multinomial regressions were used to examine the association (ENDS equally or more harmful compared to cigarettes = 1, ENDS less harmful than cigarettes = 0, and Don’t know = 2). Throughout the study, analyses were carried out using Stata 14 (StataCorp, TX, USA). 

## 3. Results

Table 1 presents the weighted summary statistics, for the full analytical sample, the smoker sample, and the nonsmoker sample. Among the adults who have perceptions of ENDS harms, 16.5% were smokers, and 15.3% had used ENDS. Furthermore, 47.8% considered ENDS to be more or equally harmful compared to cigarettes. Ad exposure measures show that 81.4% of this group of adults had seen ENDS ads, and 11.4% potentially had been exposed to magazine ENDS ads with warnings. The average age of this population was 45.1, with a household size of 2.8 and an average of 0.6 children. Race/ethnicity breakdowns show that 67.4% were White, non-Hispanic; 9.9% were Black, non-Hispanic; 6.9% were other race, non-Hispanic; 14.6% were Hispanic; and 1.2% were of two or more races. In addition, 51.8% were female and 59% were married, while 58.5% were employed, 10.9% were not employed, and 30.5% were not in the labor force. With regards to household income, 22.1% earned $24,999 or less, 34.9% earned $25,000–$74,999, and 43.1% earned $75,000 or more. For educational achievement, 12.1% had a less than high school degree, 27.4% had a high school degree, 29.4% had some college education, and 31.2% had a bachelor’s degree or higher. 

Table 1 also reports summary statistics by smoking status. The rate of ever-use of ENDS was higher among smokers (53.3%) than among non-smokers (7.8%). Smokers compared with non-smokers were less likely to consider ENDS to be equally or more harmful than cigarettes (42.1% vs 48.9%). Smokers were more likely to report ever seeing ENDS ads (90.1% vs 79.6%). Compared with non-smokers, smokers were more likely to be in the lower income and education categories, and less likely to be employed. 

Table 2 shows the associations between correlates and believing ENDS to be equally or more harmful compared to traditional cigarettes, instead of considering ENDS to be less harmful. The results from logistic regressions show that females (OR = 1.44; 95% CI: 1.24–1.68), Black, non-Hispanic adults (OR = 1.38; 95% CI: 1.06–1.81) and Hispanic adults (OR = 1.73; 95% CI: 1.33–2.55) were more likely to consider ENDS being equally or more harmful compared to cigarettes. By contrast, ever used ENDS (OR = 0.41; 95% CI: 0.32–0.52), having a household income of $75,000 or more (OR = 0.72; 95% CI: 0.57–0.91), and having a college degree or higher (OR = 0.67; 95% CI: 0.48–0.92) were significantly associated with decreased odds of considering ENDS to be equally or more harmful compared to cigarettes. Seeing ENDS ads (OR = 0.71; 95% CI: 0.58–0.86) and higher exposure to voluntary warnings in magazine ads (OR = 0.26; 95% CI: 0.07–0.98) were also associated with lowered odds of considering ENDS to be equally or more harmful than cigarettes.

Table 2 also contains results stratified by smoking status. For both smokers and non-smokers, ever used ENDS was negatively associated with considering ENDS to be equally or more harmful than cigarettes (for smokers OR = 0.49, 95% CI: 0.35-0.68; for non-smokers, OR = 0.35, 95% CI: 0.24–0.5). Having seen ENDS ads (OR = 0.71; 95% CI: 0.58–0.88) and the potential exposure to magazine ENDS ads with warnings (OR = 0.16; 95% CI: 0.04–0.77) were negatively associated with harm perception (ENDS ≥ cigarettes) among non-smokers, but not among smokers. For smokers, except for race/ethnicity, socio-demographic variables were not significantly associated with harm perceptions. Compared to White, non-Hispanic smokers, other race, non-Hispanic (OR = 2.4; 95% CI: 1.05–5.47) smokers were more likely to consider ENDS to be equally or more harmful compared to cigarettes, whereas multi-race, non-Hispanic smokers (OR = 0.27; 95% CI: 0.08–0.96) were less likely to report such a perception. Among nonsmokers, females (OR = 1.55; 95% CI: 1.31–1.84), Black (OR = 1.5; 95% CI: 1.09–2.08), and multi-race, non-Hispanic (OR = 1.68; 95% CI: 1.07–2.64), and Hispanic (OR = 1.68; 95% CI: 1.07–2.64) were more likely to perceive ENDS to be equally or more harmful compared to cigarettes. In contrast, having an income of $75,000 or more (OR = 0.75; 95% CI: 0.58–0.98) and having a college degree or above (OR = 0.63; 95% CI: 0.43–0.92) were associated with lower odds of perceiving ENDS to be equally or more harmful compared to cigarettes. The additional analyses (Appendix A Table A1) yield very similar conclusions. In particular, the potential exposure to magazine ENDS ads with warnings were negatively associated with harm perception (ENDS ≥ cigarettes) while positively associated with considering ENDS to less harmful than cigarettes among non-smokers. This pattern was not found for smokers. 

## 4. Discussion

This study aims to explore how the potential exposure to magazine ENDS ads with warnings are associated with harm perceptions after controlling for total ENDS ad exposure, ENDS ever use status, and demographic characteristics. We find that higher exposure to ENDS magazine ads with warnings was associated with a lower probability of non-smokers considering ENDS to be equally or more harmful compared to cigarettes. By contrast, this pattern was not found among smokers. 

Several potential scenarios may explain this finding. First, given that we could not distinguish exposure to ENDS ads with warnings from exposure to warnings, it is likely the estimated association captured the effects of magazine ads rather than warnings per se. In particular, the warnings evaluated in this study evaluates magazine ads with warnings that are voluntarily used by manufacturers and are smaller in size and less noticeable than the FDA-required warning statement [7]. One prior study showed that participants rarely pay attention to warnings that are embedded in an ad in a manner similar to the magazine ads with voluntary warnings observed in this study [21]. Second, it is possible that the ads with warnings appear more trustworthy for nonsmokers, and thus, mediate the effects of ads instead of the effects of warnings. As a result, we find that exposure to ENDS voluntary warning in magazine ads with warnings was positively associated with perceiving ENDS to be less harmful than cigarettes. Third, it is also possible that nonsmokers are more likely than smokers to have misperceptions of ENDS relative harmfulness, and are less likely to believe its potential benefits for smokers [27,28]. ENDS addiction warnings may help them to re-evaluate their relative harm perceptions. Future research is needed to evaluate the aforementioned competing explanations for the positive association between warning exposure and perception that ENDS are less harmful than cigarettes.

The non-significant association found for smokers is consistent with the existing literature in several aspects. One focus group study suggests that consumers consider the ENDS addiction warning to be ineffective for smokers because they have already learned about nicotine and addiction [12]. Since a large proportion of voluntary warnings (notably Blu) carried the proposed FDA warning [7] the non-significant association between exposure to ENDS magazine ads with warnings and harm perception among smokers may confirm this prior finding. 

Consistent with the existing literature [38], exposure to ENDS ads is positively associated with perceiving ENDS to be less harmful than cigarettes. Having tried ENDS is significantly associated with higher probabilities of considering ENDS as less harmful than cigarettes. In fact, although smokers compared to non-smokers were more likely to consider ENDS as less harmful than cigarettes, once ENDS ever use status was controlled for, smoking status was no longer associated with harm perceptions. Minority non-smokers were more likely to perceive ENDS to be equally or more harmful than cigarettes. This finding highlights that the misperception about relative harms is more prevalent among Black and Hispanic populations than among the White population, which may further exacerbate the existing racial/ethnic disparity in smoking and related harms. 

Gender patterns suggest that, although gender does not play a significant role in smokers’ risk perception, female nonsmokers were more likely to perceive ENDS to be equally or more harmful compared to cigarettes. This misperception potentially results from nicotine poisoning of children or voluntary warning messages about the potential harms of nicotine to pregnant women [7,39]. In fact, a recent study documents that participants tend to report greater perceived ENDS harms to unborn baby than other risk beliefs [25]. In addition, nonsmokers with higher income and education were more likely to perceive ENDS to be less harmful than cigarettes, suggesting that these factors help to form correct risk perceptions. 

This study has several limitations. Foremost, this study is observational, which limits the degree to which causal linkages can be inferred. Related, we could not distinguish exposure to ads with warnings from the exposure to warnings, which should be addressed in future studies. In addition, we only knew self-reports of ever exposure to ENDS ads, instead of exposure in the past three to six months. Therefore, we used the state-level average to better approximate recent marketing activities. Because of the lack of voluntary warning trend data in recent years, we could not utilize multiple waves of the Tobacco Products and Risk Perception surveys to conduct this study. ENDS and tobacco control policies at the local level may also shape risk perceptions, which were not controlled for in the present study. As one study suggested, harm perceptions can be evaluated using different measures (e.g., harm perception versus socio-norm perceptions); a future study with more comprehensive measures is needed [40]. In addition, we did not evaluate addiction perceptions in this study, which will be evaluated in a follow-up study. 

## 5. Conclusions

This study examined the association between the potential exposure to ENDS magazine ads with voluntary warnings and perceived relative harms of ENDS to cigarettes. The associations of other correlates, such as total ad exposure, tobacco use, and demographic characteristics, with harm perceptions, were also evaluated. We found that for nonsmokers, higher exposure to ENDS magazine ads with voluntary warnings were significantly associated with perceiving ENDS to be less harmful than cigarettes, but this pattern was not found for smokers. In summary, we did not find evidence that magazine ads with warnings increased misperceptions that ENDS are equally or more harmful than cigarettes. In addition, total ad exposure, ENDS use history, race/ethnicity, gender, education, and income also play a role in shaping harm perceptions. 

## Figures and Tables

**Table 1:** Weighted descriptive statistics—2014 Tobacco Products and Risk Perceptions Survey ^a^.

Variable Name	Full Sample (*N* = 3642)	Smokers (*N* = 865)	Non-Smokers (*N* = 2777)
Tobacco use			
Current smoker	16.5%	--	--
Ever used ENDS	15.3%	53.3%	7.8%
Harm perception			
ENDS equally or more harmful compared to cigarettes	47.8%	42.1%	48.9%
Ad with warning exposure			
Seen any ads	81.4%	90.1%	79.6%
Potential exposure to magazine ads with warnings (state-level)	11.5%	11.4%	11.5%
Socio-demographic variables			
Race/Ethnicity			
White, non-Hispanic	67.4%	64.8%	67.9%
Black, non-Hispanic	9.9%	16.1%	8.7%
Other, non-Hispanic	6.9%	5.2%	7.2%
Hispanic	14.6%	12.5%	15.0%
2+ Races, non-Hispanic	1.2%	1.4%	1.2%
Age	45.05 (0.33)	42.94 (0.60)	45.46 (0.37)
Female	51.8%	47.6%	52.6%
Married	59%	50.1%	60.7%
Household size	2.81 (0.03)	2.81 (0.07)	2.81 (0.03)
# of children <18 in household	0.59 (0.02)	0.60 (0.04)	0.59 (0.02)
Household income			
≤$29,999	22.1%	39.8%	18.6%
$30,000–$74,999	34.9%	37.8%	34.3%
≥$75,000	43.1%	22.4%	47.2%
Employment			
Unemployed	10.9%	14.5%	10.2%
Employed	58.5%	52.8%	59.7%
Not in the labor force	30.5%	32.6%	30.1%
Education			
Less than high school	12.1%	19.1%	10.7%
High school	27.4%	36.0%	25.7%
Some college	29.4%	33.7%	28.5%
Bachelor’s degree or higher	31.2%	11.3%	35.1%

Note: Percent % or Mean (SE) are reported. ^a^ Survey participants who responded with the “don’t know” option to the question, “Is using e-cigarettes less harmful, about the same level of harm, or more harmful than smoking regular cigarettes?” are excluded from this table.

**Table 2:** The association between potential exposure to ENDS magazine ads with voluntary warnings and harm perceptions ^a^.

Variable Name	All Respondents (*N* = 3642)	Smokers (*N* = 865)	Nonsmokers (*N* = 2777)
Seen any ENDS ads	0.71 *** (0.58–0.86)	0.67 (0.40–1.13)	0.71 *** (0.58–0.88)
Potential exposure to magazine ads with warnings (state-level)	0.26 * (0.07–0.98)	2.85 (0.23–35.5)	0.16 * (0.04–0.77)
Current smokers	1.07 (0.85–1.34)	--	--
Ever used ENDS	0.41 *** (0.32–0.52)	0.49 *** (0.35–0.68)	0.35 *** (0.24–0.50)
Race/ethnicity-White—non-Hispanic omitted as the reference group			
Black, non-Hispanic	1.38 * (1.06–1.81)	1.05 (0.64–1.73)	1.50 * (1.09–2.08)
Other, non-Hispanic	1.15 (0.80–1.64)	2.40 * (1.05–5.47)	1.03 (0.72–1.55)
Hispanic	1.73 *** (1.33–2.25)	1.70 ^+^ (0.96–3.02)	1.80 *** (1.33–2.42)
2+ Races, non-Hispanic	1.23 (0.80–1.90)	0.27 * (0.08–0.96)	1.68 * (1.07–2.64)
Age	0.99 (0.97–1.02)	0.96 (0.90–1.03)	1.001 (0.97–1.03)
Age squared	1.0002 (1.00–1.0005)	1.0004 (1.00–1.001)	1.0001 (1.00–1.0005)
Female	1.44 *** (1.24–1.68)	1.08 (0.77–1.52)	1.55 *** (1.31–1.84)
Married	1.07 (0.90–1.28)	1.08 (0.75–1.56)	1.05 (0.86–1.29)
Household size	1.02 (0.93–1.11)	1.08 (0.91–1.28)	1.01 (0.91–1.12)
# Children	1.10 (0.98–1.24)	0.92 (0.72–1.18)	1.13 ^+^ (0.99–1.30)
Household income—≤$24,999 omitted as the reference group			
$25,000–$74,999	0.89 (0.71–1.10)	0.69 ^+^ (0.46–1.02)	0.96 (0.74–1.24)
≥$75,000	0.72 ** (0.57–0.91)	0.68 (0.41–1.14)	0.75 * (0.58–0.98)
Employment—unemployed omitted as the reference group			
Employed	1.17 (0.87–1.57)	0.85 (0.49–1.46)	1.24 (0.88–1.75)
Not in labor force	0.99 (0.72–1.36)	1.01 (0.56–1.83)	0.99 (0.68–1.45)
Education—less than high school omitted as the reference group			
High school	0.79 (0.58–1.08)	0.96 (0.57–1.63)	0.74 (0.50–1.08)
Some college	0.76 ^+^ (0.55–1.03)	0.92 (0.54–1.56)	0.71 ^+^ (0.49–1.05)
Bachelor’s degree or higher	0.67 * (0.48–0.92)	0.67 (0.34–1.35)	0.63 * (0.43–0.92)

Note: Odds ratios and corresponding 95% confidence intervals from weighted logistic regressions. *** *p* ≤ 0.001, ** *p* ≤ 0.01, * *p* ≤ 0.05, ^+^ *p* ≤ 0.1. ENDS equally or more harmful compared to cigarettes = 1, and ENDS less harmful than cigarettes = 0. ^a^ Survey participants who responded with the “don’t know” option to the question, “Is using e-cigarettes less harmful, about the same level of harm, or more harmful than smoking regular cigarettes?” are excluded from this table.

## Appendix A

Examples of language used in warnings by different ENDS brands.

MarkTen: Rolling Stone RS1214, 31 July 2014, a two-page spread ad in the magazine cover.

“WARNING: This is not intended for use by women who are pregnant or breastfeeding, or persons with or at risk of heart disease, high blood pressure, diabetes, or taking medicine for depression or asthma. Nicotine is addictive and habit forming, and it is very toxic by inhalation. Nicotine can increase your heart rate and blood pressure and cause dizziness, nausea, and stomach pain. Inhalation of this product may aggravate existing respiratory conditions.”

Blu: Popular Mechanics, 1 November 2014, a one-page ad in the magazine.

“NOT FOR SALE TO MINORS. WARNING: This product contains nicotine derived from tobacco. Nicotine is an addictive chemical. © 2014 LOEC, Inc., blu^TM^ and blu eCigs^®^ and blu logo are trademarks of Lorillard Technologies, Inc. (photograph by Francesco Carrozzini).”

**Table A1:** The association between potential exposure to ENDS magazine ads with voluntary warnings and harm perceptions, weighted multinomial regressions.

Variables	All Respondents (*N* = 5629)			Smokers (*N* = 1322)			Nonsmokers (*N* = 4307)		
	ENDS < Cigs.	ENDS ≥ Cigs.	Don’t Know	ENDS < Cigs.	ENDS ≥ Cigs.	Don’t Know	ENDS < Cigs.	ENDS ≥ Cigs.	Don’t Know
Seen any ENDS ads	0.27 *** (0.04)	0.01 (0.04)	−0.32 *** (0.04)	0.34 ** (0.11)	0.01 (0.012)	−0.35 ** (0.11)	0.26 *** (0.04)	0.01 (0.05)	−0.32 *** (0.05)
Potential exposure to magazine ads with warnings (state-level)	0.095 ^+^ (0.050)	−0.098 ^+^ (0.060)	−0.01 (0.05)	−0.03 (0.10)	0.09 (0.10)	−0.05 (0.10)	0.12 * (0.06)	−0.14 * (0.07)	0.004 (0.061)
Current smokers	−0.07 (0.06)	−0.03 (0.07)	0.10 (0.06)	--	--	--	--	--	--
Ever used ENDS	0.60 *** (0.07)	−0.26 *** (0.08)	−0.35 *** (0.08)	0.63 *** (0.11)	−0.09 (0.11)	−0.45*** (0.09)	0.59 *** (0.095)	−0.43 *** (0.12)	−0.21 ^+^ (0.11)

Note: Marginal effects and corresponding standard errors are reported. The regressions also controlled for race/ethnicity, age, gender, marital status, household characteristics, employment status, education and income. *** *p* ≤ 0.001, ** *p* ≤ 0.01, * *p* ≤ 0.05, ^+^ *p* ≤ 0.1. ENDS equally or more harmful compared to cigarettes = 1, ENDS less harmful than cigarettes = 0, and Don’t know = 2.
